# Psychosocial Variables Related to Weight-Related Self-Stigma in Physical Activity among Young Adults across Weight Status

**DOI:** 10.3390/ijerph17010064

**Published:** 2019-12-20

**Authors:** Xavier C. C. Fung, Amir H. Pakpour, Ya-Ke Wu, Chia-Wei Fan, Chung-Ying Lin, Hector W. H. Tsang

**Affiliations:** 1 Department of Rehabilitation Sciences, The Hong Kong Polytechnic University, Hung Hom, Hong Kong, China; xavierfungzzz@gmail.com (X.C.C.F.); Hector.Tsang@polyu.edu.hk (H.W.H.T.); 2 Social Determinants of Health Research Center, Research Institute for Prevention of Non-Communicable Diseases, Qazvin University of Medical Sciences, Shahid Bahounar BLV, Qazvin 3419759811, Iran; pakpour_amir@yahoo.com; 3 Department of Nursing, School of Health and Welfare, Jönköping University, SE-551 11 Jönköping, Sweden; 4 School of Nursing, The University of North Carolina at Chapel Hill, Chapel Hill, NC 27599, USA; yakew@email.unc.edu; 5 Department of Occupational Therapy, AdventHealth University, Orlando, FL 32803, USA; chia-wei.fan@ahu.edu

**Keywords:** overweight, stigma, physical activity, the theory of planned behavior

## Abstract

A healthy lifestyle with sufficient physical activity (PA) can contribute to weight management. Yet, many people do not maintain a healthy lifestyle. To explain PA, we propose a model that incorporates the Theory of Planned Behavior (TPB) with weight-related self-stigma. We recruited 325 young adults to complete questionnaires regarding their physical activities, weight-related self-stigma, and TPB factors. We used structural equation modeling to examine the model fit and the path invariance across weight groups. The model showed excellent model fit, but path invariance was not supported. Weight-related self-stigma significantly explained the perceived behavioral control, behavioral intention, and engagement of PA. People without overweight and people with overweight have different considerations for PA. Weight-related self-stigma is important for PA as well. To promote a healthy lifestyle, healthcare providers should provide different suggestions or interventions that suit their patients’ weight-related concerns.

## 1. Introduction

Obesity is a worldwide health concern. A global study of 188 countries revealed that over one-third of both men and women are overweight or obese [1]. In Hong Kong, specifically, the prevalence of overweight had increased from 38.8% in 2004 to 50% in 2015 among people aged 15–84 [2,3]. Research has shown that individuals with overweight or obesity are at an increased risk of cardiovascular disease and lower quality of life [4,5]. Therefore, healthcare providers are eager to raise people’s awareness about the importance of engaging in weight management behaviors that address weight-related health concerns.

Physical activity (PA) is an important factor in healthy weight management [6,7]. For adults, the World Health Organization (WHO) recommends at least 150 min of moderately intense aerobic PA per week [8]. Moreover, low levels of PA lead to weight gain [9]. The global prevalence of insufficient PA is around 30%, and the prevalence is higher in high–income countries [10]. Many adults in Hong Kong do not achieve sufficient PA. A survey indicated that 34% of Hong Kong adults report rarely or never exercising in the past six months [11]. Tackling the issues of insufficient PA is critical for Hong Kong populations to achieve better weight management.

The Theory of Planned Behavior (TPB) explains and predicts specific behaviors [12], including help-seeking, medication adherence, PA, and food intake [13,14,15,16]. The TPB incorporates five constructs: attitudes, subjective norms, perceived behavioral control (PBC), behavioral intentions, and the particular behavior itself. Attitude refers to the extent to which a person feels and evaluates the particular behavior with its outcomes. Subjective norm refers to the perceived social approval/disapproval of engaging in that behavior within a person’s social context. PBC refers to the perceived capability of an individual to perform a specific behavior. Behavioral intention refers to the motivation and formulation of an implementation plan, for example, when and how to perform the specific behavior [12].

TPB hypothesizes that attitude, subjective norm, and PBC contribute to behavioral intention, and that the behavior itself is predicted by both behavioral intention and PBC [12]. Although TPB has been used for assessing PA, there are contradictions between studies. In one study conducted in Canadian adolescents, TPB successfully explained the behavior of PA [17], but in another study in US women in a weight-loss program, the TPB constructs PBC and intention did not explain PA levels [18]. Current literature also shows the applicability of TPB to PA. Specifically, TPB factors (except for subjective norm on males) and self-efficacy significantly explained leisure time PA engagement [18], and another study found that TPB factors better explained the variance of behaviors based on volition (e.g., dancing) rather than that of required daily trainings (e.g., running, swimming, and team sports) [19].

To the best of our knowledge, TPB studies related to weight management behaviors have been conducted mainly in Western countries, but evidence from East Asian countries is lacking. Cultural differences also may influence the TPB factors, such as subjective norm and PBC [20,21]. For example, Lee et al. [21] suggested that cultural self-construal, how an individual perceives one’s independent ability and interpersonal connection within the culture, may be associated with PBC (independent) and subjective norm (interpersonal). Given the divergence of past TPB research results and the limited TPB research in East Asian countries, the link between TPB and PA for Hong Kong populations requires additional research.

Moreover, healthcare providers should consider cultural differences when they want to intervene or study weight management and stigma perception. Indeed, a study comparing Polish (Western) and Vietnamese (Eastern) people in the conception of body shape found that Vietnamese showed higher scores of weight-related self-stigma than Polish [22], possibly due to collectivism [23,24], a culture which integrated people into groups and put emphasis on cohesion and loyalty [25]. Previous TPB studies suggested that there was cultural impact on Hong Kong Chinese, for example, breastfeeding, use of traditional Chinese medicine [26,27]. Particularly, the study on traditional Chinese medicine indicated the greater influence of subjective norm was due to collectivistic culture [27]. Additionally, engagement in PA was found to be different between Eastern and Western countries [9]. Hence, research in Hong Kong is needed.

The TPB has been criticized for its parsimony, however, thus warranting its expansion to include potentially related factors [28,29]. In addition to the TPB, weight-related self-stigma may affect PA. Weight stigma is the devaluation and discrimination of people due to their body weight. Individuals with overweight/obesity are often exposed to a high risk of being stigmatized by others [30]. These negative, direct life experiences or attitudes may cause these individuals to incur weight-related self-stigma, which is the internalization of these biases that promote self-devaluation [31]. Weight bias could also become internalized in response to the cultural value of thinness and derogation of overweight and obesity transmitted via media (e.g., TV shows and fitness advertisements) [32].

Several studies have suggested that self-stigma may lead to poor health conditions. Major, Eliezer, and Rieck [33] found that concern about weight stigma increased stress and reduced self-control in stigmatized individuals. Weight-related self-stigma may also potentially influence PBC because weight-related self-stigma is associated with poor self-concept (the belief about oneself, such as characteristics and abilities) [34]. For example, a study [35] found that girls with obesity showed lower self-concept, including their competence and behavioral conduct, compared with girls without overweight. Furthermore, the self-concept was failed to rebound in girls who were formerly overweight, due to the effect of lingering self-stigma [35]. Another study found that individuals with obesity were more likely to report lower self-concept when they were being stigmatized [36]. Thus, it is possible that weight-related self-stigma may impact on PBC, but there is no direct evidence to account for this relationship. Moreover, the stress induced by weight stigma may, in turn, reduce the level of PA and promote further weight gain [37]. Furthermore, a negative association was also found between weight-related self–stigma and PA [38]. Therefore, we propose to add weight-related self-stigma as a potential variable into TPB to serve as the extended TPB model for weight-related outcomes.

The present study aimed to investigate whether the extended TPB—the original TPB incorporated with weight-related self-stigma—could explain the PA of young adults in Hong Kong. As mentioned, people with overweight may have different feelings, such as their self-concept and body image, from those without overweight [35,36]. Thus, we were also interested in determining whether people with different weight status (without overweight vs. overweight) fit with the model similarly.

## 2. Materials and Methods

### 2.1. Participants and Procedures

We recruited 325 young adults from The Hong Kong Polytechnic University with the use of convenience sampling. A priori sample size of 300 was determined as sufficient in statistical power according to prior studies investigating similar topics (i.e., studies on TPB and PA) [39,40,41]. With the cooperation of several faculty members in the Departments of Rehabilitation Science and Mechanical Engineering, 450 university students were invited to participate in this study. These faculties allowed several research assistants to use the final 20 min of a lecture period to explain the study to the students in attendance. Students interested in the study then were asked to log in to a *Google Form* through a QR code (a type of two-dimensional barcode) to complete a series of questionnaires. The Ethics Review Board of the university approved the study prior to collecting the data. Electronic informed consent was obtained before the participants began to answer the questionnaires.

Participants were asked to report their current height and weight and were classified as “without overweight” or “with overweight” based on their body mass index (BMI). We adopted the BMI cutoff from the World Health Organization (WHO) [42]. We classified Asian participants with a BMI of 18.5–22.9 kg/m^2^ as without overweight, and those with equal to/greater than 23 kg/m^2^ as overweight. We set the inclusion criteria as follows: (1) 18 to 30 years old [43], (2) able to read and understand Chinese, and (3) agree to participate. Participants were excluded if they had self-reported neurological illness (e.g., stroke, autism), functional disability (e.g., blindness), or any type of psychosis or intellectual disability that would make it difficult to complete the online surveys.

### 2.2. Measures

#### 2.2.1. Physical Activity

*International Physical Activity Questionnaire (IPAQ)*. We used the Chinese version of the IPAQ, which is a self-reported questionnaire that measures the amount of PA in the past week [44]. A sample item is “During the last seven days, on how many days did you do vigorous physical activities?” [44]. The item scores on the questionnaire were converted into the metabolic equivalent of task (MET) according to the time spent on different levels of PA (MET = 1 for sitting, 3.3 for walking, 4 for moderate PA, 8 for vigorous PA) [44]. For example, if a person walks 40 min every day, the PA should be 3.3 × 40 min × 7 days = 924 METs. Thus, a higher number indicated a higher level of PA. The test–retest reliability of the IPAQ was satisfactory, as was the intraclass correlation coefficient (ICC) = 0.79 [45].

#### 2.2.2. TPB Factors

To assess the associations between PA and TPB factors, we used another questionnaire to assess four of the constructs in TPB: attitude, subjective norm, PBC, and behavioral intention.

*Attitude*. We used eight 7-point items measured on a semantic differential scale for PA [16]. Bipolar adjective pairs were used (pleasant–unpleasant, good–bad, beneficial–harmful, wise–foolish, correct–incorrect, enjoyable–unenjoyable, satisfying–unsatisfying, useful–useless) with the item stem of “For me to exercise at least 30 min, three days per week is ____.” A higher score indicated a more positive attitude about the behavior [16]. The internal consistency (Cronbach’s α) was 0.90 for the attitude toward PA.

*Subjective Norm*. We used three 7-point items to assess the subjective norms towards PA [16]. Sample items were “People who are important to me would approve of me every day,” in which the blank was filled in with “exercising/exercise at least 30 min, at least three days per week.” Higher scores indicated higher levels of the subjective norm. The internal consistency (Cronbach’s α) was 0.82 for the subjective norm toward PA.

*Perceived Behavioral Control (PBC)*. We used four 7-point items to assess PBC towards PA [46]. Sample items were “How much personal control do you feel you have over whether you in the next week?” The blank was filled in with “exercising/exercise at least 30 min, at least three days per week.” Higher scores indicated higher levels of PBC. The internal consistency (Cronbach’s α) was 0.95 for the PBC toward PA.

*Behavioral Intention*. We used three 7-point items to assess the behavioral intentions for PA [47]. Sample items were “I plan to from now on,” in which the blank was filled in with “exercising/exercise at least 30 min, at least three days per week.” Higher scores indicated higher levels of behavioral intention. The internal consistency (Cronbach’s α) was 0.97 for the intention toward PA.

#### 2.2.3. Weight-Related Self-Stigma

*Weight Bias Internalization Scale (WBIS)*. We used the WBIS to measure participants’ weight-related self-stigma [31]. All 11 items were rated on a 5-point scale from 1 (strongly disagree) to 5 (strongly agree); a higher score indicated a higher level of weight-related self-stigma. We used the Chinese version of WBIS with satisfactory psychometric properties [48]. The internal consistency (Cronbach’s α) of the WBIS was 0.91.

### 2.3. Data Analysis

Given that each of our models consists of six observed variables (attitude, subjective norm, PBC, behavioral intention, weight-related self-stigma, PA) with eight path coefficients needed to be estimated, the model would have 13 degrees of freedom based on the following equation: ½ × number of parameters × (number of parameters + 1) — free parameters [49]. Using 13 degrees of freedom accompanied by type I error at 0.05, power at 0.8, null root mean square error of approximation at 0.08, and alternative root mean square error of approximation at 0, the sample size required for adequate power was estimated to be ~216 [50]. Therefore, 325 participants were sufficient to provide adequate statistical power.

We used Pearson’s correlation to examine the relationships among TPB factors, weight-related self-stigma, and PA. We conducted structural equation modeling (SEM) with multiple group analysis to examine the fit of the proposed PA model and tested the path invariance across participants with overweight and those without overweight.

**Figure 1:**
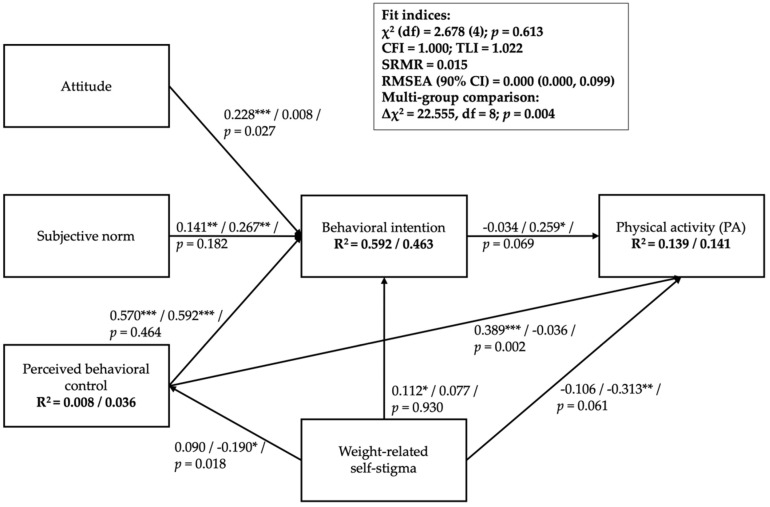
Multiple group analysis of the Theory of Planned Behavior model incorporated with weight-related self-stigma on physical activity. Note: * *p* < 0.05; ** *p* < 0.01; *** *p* < 0.001; Path coefficients (without overweight/overweight/significance test between groups); Lower score on PA indicates less PA engagement. PA, physical activity; CFI, comparative fit index; SRMR, root mean square residual; RMSEA, root mean square error of approximation.

We applied the maximum likelihood estimator to the model and applied full information maximum likelihood estimation for missing values. The proposed model is shown in Figure 1. All the factors were treated as observed variables. Specifically, we used summated scores to represent each factor in the extended TPB (e.g., WBIS total score to represent the observed weight-related self-stigma). We checked the multiple group SEMs for their fitness on the proposed model using the following indices: comparative fit index (CFI), Tucker–Lewis index (TLI), root mean square error of approximation (RMSEA), and the standardized root mean square residual (SRMR). We used the χ^2^ test to determine the fit of models. The models with nonsignificant χ^2^—CFI and TLI > 0.9, RMSEA and SRMR <0.08—were considered acceptable; that is, the proposed models were supported by the data.

After ensuring that the PA model was supported, we used the χ^2^ difference (Δχ^2^) test to check for path invariance for the model. A nonsignificant χ^2^ difference test indicated path invariance across groups. We first constrained all path coefficients as being equal across the two groups (i.e., overweight and without overweight) and tested whether the model with all constrained path coefficients was significantly different from the model without any constraints. If the two models were not significantly different, the path invariance was supported for all the paths. If the two models were significantly different, we tested the path invariance for each path coefficient.

We performed analyses using SPSS 23.0 (SPSS Inc., Chicago, IL, USA), except for SEMs, which we performed using R software (R Foundation for Statistical Computing, Vienna, Austria.) with the latent variable analysis (lavaan) package [51].

## 3. Results

Table 1 displays the demographics and scores on the instruments for all the participants. Briefly, the mean age was 21.6 (standard deviation [SD] = 2.95), the mean BMI was 22.39 (SD = 4.03), and more than half of the participants were female (61.2%). Table 2 displays the correlation among TPB factors, weight-related self-stigma, and PA.

The PA model (Figure 1) had excellent model fit: nonsignificant χ^2^ (χ^2^ (df) = 2.678 [4]; *p* = 0.613), CFI = 1.000, TLI = 1.022, SRMR = 0.015, and RMSEA = 0.000. However, the significance of the path coefficients differed between the two groups. For the non-overweight group, attitude, subjective norm, PBC, and weight-related self-stigma were significantly associated with behavioral intention; only PBC was significantly associated with PA. Attitude, subjective norm, PBC, and weight-related self-stigma together explained 59.2% of the variance in behavioral intention; weight-related self-stigma, PBC, and behavioral intention together explained 13.9% of the variance in PA. For the overweight group, only the subjective norm and PBC were significantly associated with behavioral intention; weight-related self-stigma was negatively associated with PA; behavioral intention was positively associated with PA; weight-related self-stigma was negatively associated with PBC. Attitude, subjective norm, PBC, and weight-related self-stigma together explained 46.3% of the variance in behavioral intention; 14.1% of the variance in PA was explained by weight-related self-stigma, PBC, and behavioral intention; weight-related self-stigma explained 3.6% of the variance in PBC.

The χ^2^ difference test further indicated a significant difference between constrained and unconstrained PA models (Δχ^2^ = 22.555, df = 8; *p* = 0.004; Table A1). We conducted additional path invariance tests by constraining only one of the paths each time and compared with the unconstrained model. Results showed that the relationships between PBC and PA (Δχ^2^ = 9.210, df = 1; *p* = 0.002), between attitude and intention (Δχ^2^ = 4.911, df = 1; *p* = 0.027), and between weight-related self-stigma and PBC (Δχ^2^ = 5.570, df = 1; *p* = 0.018) were significantly different across weight groups.

## 4. Discussion

We examined the weight management behaviors (i.e., PA) using a model that incorporates TPB with weight-related self-stigma. The PA model demonstrated excellent model fit. However, relationships in the PA model varied across weight groups, and the path invariance was not supported for all path coefficients. Specifically, the relationship between PBC and PA, between attitude and intention, and between weight-related self-stigma and PBC were significantly different across weight groups.

In the PA model, attitude was significantly associated with intention in the group without overweight, but not in the group with overweight; PBC was significantly associated with PA in the group without overweight, but not in the group with overweight. Therefore, attitude and PBC might not be the factors accounting for the intention toward PA and the PA engagement of participants with overweight in our sample. One possible explanation is the perceived barriers experienced by the participants with overweight. Regarding the relationship between attitude and intention to PA, previous work has found that individuals with overweight were concerned more about their body image and experienced higher social anxiety than their counterparts, which resulted in unwillingness to perform PA [52]. A previous study similarly found that as people received more criticism on PA due to their weight, they reported lower enjoyment in PA and showed avoidance of PA [53].

Regarding the relationship between PBC and intention to PA, peer victimization might be one of the barriers [54]. Individuals with excess weight may perceive fewer opportunities, may have less support, and not be welcomed by others; these uncontrollable external barriers may deter people from engaging in PA [54]. Individuals with overweight reported more barriers than individuals without overweight about not being good at PA (i.e., feeling like they are the weakest ones) [55]. Moreover, Rech et al. [56] suggested that adults with overweight simply lacked confidence in performing PA. Therefore, social environmental factors may interfere with the relationships between attitude and PA intention, and between PBA and PA intention.

We also found that weight-related self-stigma was significantly associated with PBC, behavioral intention, and outcome behaviors related to PA. Our findings aligned with a study [57] that found a negative relationship between life stressors and PBC. Specifically, self-stigma in our study can be viewed as a type of life stressor; thus, individuals with self-stigma, especially those who have experienced self-devaluation by their labels (e.g., weak or useless), may feel incompetent and powerless [31,34]. These factors could explain why weight-related self-stigma was negatively associated with PBC in the PA model for the overweight group only. Also, weight-related self-stigma was negatively associated with PA, perhaps due to diminished self-esteem and self-efficacy [58]. Individuals who are self-stigmatized may have less motivation for weight management [58]. This so-called “why try” effect may cancel out an individual’s original behavioral intention [58].

The present study had some limitations. First, we used a cross-sectional design; thus, causal relationships could not be concluded. Longitudinal studies should be conducted to corroborate our findings in both models. Second, all of the measurements were self-reported and might not provide accurate responses due to social desirability. Third, the representativeness of our sample was weak given that all the participants were recruited from the same university. In addition, there are other potential weight-related variables, such as self-perceived weight status, that may be a useful addition to the model to explain additional variance in PA and TPB constructs [59]. Future studies should investigate other relevant variables that could be incorporated with TPB model. Last, as we only investigated young adults in this study, the generalizability might be limited. For example, a study found that older adults put more emphasis on controllability regarding people who are stigmatized and more likely to report that the stigmatized conditions can be changed [60], and another study found that older adults usually have lower level of HIV-related stigma compared with young adults [61].

## 5. Conclusions

Our study examined the extended TPB model of PA, which we expanded by adding weight-related self-stigma, to explain behaviors of PA. We found that the PA model had excellent fit. However, the PA model showed significant path differences between non-overweight and overweight groups, suggesting that there were different considerations between the two groups when they plan to engage in PA. Nevertheless, the important role of weight-related self-stigma was found in the model for adults with overweight.

## Figures and Tables

**Table 1:** Demographic information among participants.

	Participants (*n* = 325)
Gender, n (%)	
Male	126 (38.8%)
Female	199 (61.2%)
Age (years), *M* (SD)	21.6 (2.95)
Body Mass Index, *M* (SD)	22.39 (4.03)
Weight status, n (%)	
Without overweight	221 (68%)
Overweight	104 (32%)
International Physical Activity Questionnaire, *M* (SD)	2008.17 (2144.41)
Weight Bias Internalization Scale, *M* (SD)	28.92 (9.58)
Theory of Planned Behavior factors, *M* (SD)	
Attitude toward PA	82.69 (13.94)
Subjective norm toward PA	47.28 (26.17)
Perceived behavioral control toward PA	59.28 (27.00)
Behavioral intention toward PA	62.21 (27.29)

*M* = mean; SD = standard deviation; PA = physical activity.

**Table 2:** Correlation matrix among Theory of Planned Behavior factors, weight-related self-stigma, physical activity, and healthy eating.

Variables	r				
	2.	3.	4.	5.	6.
1. PA	0.20 ***	−0.02	0.30 ***	0.22 ***	−0.13 *
2. Attitude toward PA	--	0.13 *	0.50 ***	0.46 ***	−0.08
3. Subjective norm toward PA		--	0.20 ***	0.34 ***	0.31 ***
4. PBC toward PA			--	0.68 ***	0.05
5. Intention toward PA				--	0.15 **
6. Weight-related self–stigma					--

PA = physical activity; PBC = perceived behavioral control. * *p* < 0.05; ** *p* < 0.01; *** *p* < 0.001.

## Appendix A

**Table A1:** Path invariance of PA model across weight groups (without overweight vs. with overweight).

Fit Index	Model 1a	Model 2b
χ^2^ (df)	2.678 (4)	25.233 (12) *
Comparative fit index	1.000	0.970
Tucker–Lewis index	1.022	0.925
RMSEA	0.000	0.082
90% CI of RMSEA	0.000; 0.099	0.036; 0.127
SRMR	0.015	0.047
Akaike Information Criterion	16680.369	16686.924

PA = physical activity; RMSEA = root mean square error of approximation; SRMR = standardized root mean square residual; Model 1a = Unconstrained model of PA model (see Figure 1); that is, freely estimated path coefficients across weight groups; Model 1b = PA model with all path coefficients constrained to be equal across weight groups. * *p* < 0.05.

## References

[1] Ng M., Fleming T., Robinson M., Thomson B., Graetz N., Margono C., Mullany E.C., Biryukov S., Abbafati C., Abera S.F. (2014). Global, regional, and national prevalence of overweight and obesity in children and adults during 1980–2013: A systematic analysis for the Global Burden of Disease Study 2013. Lancet.

[2] Centre for Health Protection, Department of Health Report of Population Health Survey 2014/2015. https://www.chp.gov.hk/files/pdf/dh_hps_2014_15_full_report_eng.pdf.

[3] Centre for Health Protection, Department of Health; Department of Community Medicine, University of Hong Kong Population Health Survey 2003/2004. https://www.chp.gov.hk/files/pdf/report_on_population_health_survey_2003_2004_en.pdf.

[4] Reilly J.J., Methven E., McDowell Z.C., Hacking B., Alexander D., Stewart L., Kelnar C.J.H. (2003). Health consequences of obesity. Arch. Dis. Child..

[5] Wong P.C., Hsieh Y.-P., Ng H.H., Kong S.F., Chan K.L., Au T.Y.A., Lin C.-Y., Fung X.C.C. (2019). Investigating the self-stigma and quality of life for overweight/obese children in Hong Kong: A preliminary study. Child Indic. Res..

[6] World Health Organization Obesity and Overweight. https://www.who.int/news-room/fact-sheets/detail/obesity-and-overweight.

[7] Bryant E.J., Caudwell P., Hopkins M.E., King N.A., Blundell J.E. (2012). Psycho-markers of weight loss. The roles of TFEQ Disinhibition and Restraint in exercise-induced weight management. Appetite.

[8] World Health Organization Global Recommendations on Physical Activity for Health 18–64 Years Old. https://www.who.int/dietphysicalactivity/physical-activity-recommendations-18-64years.pdf.

[9] Lin Y.-C., Fung X.C.C., Tsai M.-C., Strong C., Hsieh Y.-P., Lin C.-Y. (2019). Insufficient physical activity and overweight: Does caregiver screen-viewing matter?. J. Child Fam. Stud..

[10] Guthold R., Stevens G.A., Riley L.M., Bull F.C. (2018). Worldwide trends in insufficient physical activity from 2001 to 2016: A pooled analysis of 358 population-based surveys with 1.9 million participants. Lancet Glob. Health.

[11] Hong Kong Institute of Asia-Pacific Studies at CUHK Survey Findings on Attitudes Towards Physical Exercises in Hong Kong. http://www.cpr.cuhk.edu.hk/en/press_detail.php?id=2166&t=survey-findings-on-attitudes-towards-physical-exercises-in-hong-kong-released-by-hong-kong-institute-of-asia-pacific-studies-at-cuhk.

[12] Ajzen I. (1991). The Theory of Planned Behavior. Organ. Behav. Hum. Decis. Process..

[13] Beville J.M., Umstattd Meyer M.R., Usdan S.L., Turner L.W., Jackson J.C., Lian B.E. (2014). Gender differences in college leisure time physical activity: Application of the Theory of Planned Behavior and integrated behavioral model. J. Am. Coll. Health.

[14] Guillaumie L., Godin G., Vezina-Im L.-A. (2010). Psychosocial determinants of fruit and vegetable intake in adult population: A systematic review. Int. J. Behav. Nutr. Phys. Act..

[15] Lin C.-Y., Oveisi S., Burri A., Pakpour A.H. (2017). Theory of Planned Behavior including self-stigma and perceived barriers explain help-seeking behavior for sexual problems in Iranian women suffering from epilepsy. Epilepsy Behav..

[16] Lin C.-Y., Updegraff J.A., Pakpour A.H. (2016). The relationship between the Theory of Planned Behavior and medication adherence in patients with epilepsy. Epilepsy Behav..

[17] Plotnikoff R.C., Lubans D.R., Costigan S.A., Trinh L., Spence J.C., Downs S., McCargar L. (2011). A test of the Theory of Planned Behavior to explain physical activity in a large population sample of adolescents from Alberta, Canada. J. Adolesc. Health.

[18] Gardner R.E., Hausenblas H.A. (2004). Understanding exercise and diet motivation in overweight women enrolled in a weight-loss program: A prospective study using the Theory of Planned Behavior. J. Appl. Soc. Psychol..

[19] Scott E.J., Eves F.F., Hoppé R., French D.P. (2010). Dancing to a different tune: The predictive utility of the theory of planned behaviour when the behaviour is constrained. Psychol. Sport Exerc..

[20] Hassan L.M., Shiu E., Parry S. (2016). Addressing the cross-country applicability of the Theory of Planned Behaviour (TPB): A structured review of multi-country TPB studies. J. Consum. Behav..

[21] Lee H., Ebesu Hubbard A.S., O’Riordan C.K., Kim M.-S. (2006). Incorporating culture into the theory of planned behavior: Predicting smoking cessation intentions among college students. Asian J. Commun..

[22] Lipowska M., Truong Thi Khanh H., Lipowski M., Różycka-Tran J., Bidzan M., Ha T. (2019). The Body as an Object of Stigmatization in Cultures of Guilt and Shame: A Polish–Vietnamese Comparison. Int. J. Environ. Res. Public. Health.

[23] Trinh L., Khanh H. (2019). Happy people: Who are they? A pilot indigenous study on conceptualization of happiness in Vietnam. Health Psychol. Rep..

[24] Różycka-Tran J., Ha T.T.K., Cieciuch J., Schwartz S.H. (2017). Universals and specifics of the structure and hierarchy of basic human values in Vietnam. Health Psychol. Rep..

[25] Hofstede G. (2011). Dimensionalizing Cultures: The Hofstede Model in Context. Online Read. Psychol. Cult..

[26] Dodgson J.E., Henly S.J., Duckett L., Tarrant M. (2003). Theory of Planned Behavior-Based Models for Breastfeeding Duration Among Hong Kong Mothers. Nurs. Res..

[27] Rochelle T.L., Shardlow S.M., Ng S.H. (2015). Using the Theory of Planned Behaviour to Explain Use of Traditional Chinese Medicine among Hong Kong Chinese in Britain. Evid. Based Complement. Alternat. Med..

[28] Ajzen I. (2015). The Theory of Planned Behaviour is alive and well, and not ready to retire: A commentary on Sniehotta, Presseau, and Araújo-Soares. Health Psychol. Rev..

[29] Conner M., Armitage C.J. (1998). Extending the Theory of Planned Behavior: A Review and Avenues for Further Research. J. Appl. Soc. Psychol..

[30] Puhl R.M., Heuer C.A. (2009). The stigma of obesity: A review and update. Obesity.

[31] Durso L.E., Latner J.D. (2008). Understanding self-directed stigma: Development of the Weight Bias Internalization Scale. Obesity.

[32] Frederick D.A., Saguy A.C., Sandhu G., Mann T. (2016). Effects of competing news media frames of weight on antifat stigma, beliefs about weight and support for obesity-related public policies. Int. J. Obes..

[33] Major B., Eliezer D., Rieck H. (2012). The psychological weight of weight stigma. Soc. Psychol. Personal. Sci..

[34] Tomiyama A.J., Carr D., Granberg E.M., Major B., Robinson E., Sutin A.R., Brewis A. (2018). How and why weight stigma drives the obesity ‘epidemic’ and harms health. BMC Med..

[35] Mustillo S.A., Hendrix K.L., Schafer M.H. (2012). Trajectories of Body Mass and Self-Concept in Black and White Girls: The Lingering Effects of Stigma. J. Health Soc. Behav..

[36] Carr D., Friedman M.A. (2005). Is Obesity Stigmatizing? Body Weight, Perceived Discrimination, and Psychological Well-Being in the United States. J. Health Soc. Behav..

[37] Mouchacca J., Abbott G.R., Ball K. (2013). Associations between psychological stress, eating, physical activity, sedentary behaviours and body weight among women: A longitudinal study. BMC Public Health.

[38] Pearl R.L., Puhl R.M., Dovidio J.F. (2015). Differential effects of weight bias experiences and internalization on exercise among women with overweight and obesity. J. Health Psychol..

[39] Hales D., Evenson K.R., Wen F., Wilcox S. (2010). Postpartum physical activity: Measuring Theory of Planned Behavior constructs. Am. J. Health Behav..

[40] Santina T., Godin G., Gagné C., Guillaumie L. (2017). Psychosocial determinants of physical activity at school among Lebanese children: An application of the planned behavior theory. J. Phys. Educ. Sport.

[41] Williams S.L., Michie S., Dale J., Stallard N., French D.P. (2015). The effects of a brief intervention to promote walking on Theory of Planned Behavior constructs: A cluster randomized controlled trial in general practice. Patient Educ. Couns..

[42] World Health Organization The Asia–Pacific Perspective: Redefining Obesity and its Treatment. http://www.wpro.who.int/nutrition/documents/docs/Redefiningobesity.pdf.

[43] Yen J.-Y., Lin H.-C., Chou W.-P., Liu T.-L., Ko C.-H. (2019). Associations Among Resilience, Stress, Depression, and Internet Gaming Disorder in Young Adults. Int. J. Environ. Res. Public. Health.

[44] Booth M. (2000). Assessment of physical activity: An international perspective. Res. Q. Exerc. Sport.

[45] Macfarlane D.J., Lee C.C.Y., Ho E.Y.K., Chan K.L., Chan D.T.S. (2007). Reliability and validity of the Chinese version of IPAQ (short, last 7 days). J. Sci. Med. Sport.

[46] Latimer A.E., Martin Ginis K.A. (2005). The Theory of Planned Behavior in prediction of leisure time physical activity among individuals with spinal cord injury. Rehabil. Psychol..

[47] Kothe E.J., Mullan B.A., Butow P. (2012). Promoting fruit and vegetable consumption. Testing an intervention based on the theory of planned behaviour. Appetite.

[48] Pakpour A.H., Tsai M.-C., Lin Y.-C., Strong C., Latner J.D., Fung X.C.C., Lin C.-Y., Tsang H.W.H. (2019). Psychometric properties and measurement invariance of the Weight Self-Stigma Questionnaire and Weight Bias Internalization Scale in children and adolescents. Int. J. Clin. Health Psychol..

[49] Bollen K.A. (1989). Structural Equations with Latent Variables.

[50] Preacher K.J., Coffman D.L. Computing Power and Minimum Sample Size for RMSEA. http://quantpsy.org/rmsea/rmsea.htm.

[51] Rosseel Y., Oberski D., Byrnes J., Vanbrabant L., Savalei V., Merkle E., Hallquist M., Rhemtulla M., Katsikatsou M., Barendse M. Package “Lavaan”. https://cran.r-project.org/web/packages/lavaan/lavaan.pdf.

[52] Fernández I., Canet O., Giné-Garriga M. (2017). Assessment of physical activity levels, fitness and perceived barriers to physical activity practice in adolescents: Cross-sectional study. Eur. J. Pediatr..

[53] Faith M.S., Leone M.A., Ayers T.S., Heo M., Pietrobelli A. (2002). Weight criticism during physical activity, coping skills, and reported physical activity in children. Pediatrics.

[54] Gray W.N., Janicke D.M., Ingerski L.M., Silverstein J.H. (2008). The impact of peer victimization, parent distress and child depression on barrier formation and physical activity in overweight youth. J. Dev. Behav. Pediatr..

[55] Deforche B.I., De Bourdeaudhuij I.M., Tanghe A.P. (2006). Attitude toward physical activity in normal-weight, overweight and obese adolescents. J. Adolesc. Health.

[56] Rech C., Camargo E., Almeida M., Bronoski R., Okuno N., Reis R. (2016). Barriers for physical activity in overweight adults. Rev. Bras. Ativ. Física Saúde.

[57] Louis W.R., Chan M.K.-H., Greenbaum S. (2009). Stress and the Theory of Planned Behavior: Understanding Healthy and Unhealthy Eating Intentions. J. Appl. Soc. Psychol..

[58] Corrigan P.W., Larson J.E., Rüsch N. (2009). Self-stigma and the “why try” effect: Impact on life goals and evidence-based practices. World Psychiatry.

[59] Lin Y.-C., Latner J.D., Fung X.C.C., Lin C.-Y. (2018). Poor health and experiences of being bullied in adolescents: Self-perceived overweight and frustration with appearance matter. Obesity.

[60] Krendl A.C., Wolford G. (2013). Cognitive decline and older adults’ perception of stigma controllability. J. Gerontol. B Psychol. Sci. Soc. Sci..

[61] Emlet C.A., Brennan D.J., Brennenstuhl S., Rueda S., Hart T.A., Rourke S.B. (2015). The impact of HIV-related stigma on older and younger adults living with HIV disease: Does age matter?. AIDS Care.

